# Signature Proteins in Small Extracellular Vesicles of Granulocytes and CD4^+^ T-Cell Subpopulations Identified by Comparative Proteomic Analysis

**DOI:** 10.3390/ijms251910848

**Published:** 2024-10-09

**Authors:** Sara Vázquez-Mera, Pablo Miguéns-Suárez, Laura Martelo-Vidal, Sara Rivas-López, Lena Uller, Susana B. Bravo, Vicente Domínguez-Arca, Xavier Muñoz, Francisco J. González-Barcala, Juan J. Nieto Fontarigo, Francisco J. Salgado

**Affiliations:** 1 BioLympho Research Group, Department of Biochemistry and Molecular Biology, Faculty of Biology-Biological Research Centre (CIBUS), Universidade de Santiago de Compostela, 15782 Santiago de Compostela, Spain; s.vazquez.mera@usc.es (S.V.-M.); pablo.miguens@rai.usc.es (P.M.-S.); laura.martelo@rai.usc.es (L.M.-V.); sara.rivas.lopez0@rai.usc.es (S.R.-L.); juanjose.nieto.fontarigo@usc.es (J.J.N.F.); franciscojavier.salgado@usc.es (F.J.S.); 2 Translational Research in Airway Diseases Group (TRIAD), Health Research Institute of Santiago de Compostela (IDIS), 15706 Santiago de Compostela, Spain; 3 Department of Experimental Medical Science, Lund University, 22362 Lund, Sweden; lena.uller@med.lu.se; 4 Proteomic Service, Health Research Institute of Santiago de Compostela (IDIS), 15706 Santiago de Compostela, Spain; susana.belen.bravo.lopez@sergas.es; 5 Biophysics and Interfaces Group, Applied Physics Department, Faculty of Physics, Universidade de Santiago de Compostela, 15782 Santiago de Compostela, Spain; 6 Centro de Investigación en Red de Enfermedades Respiratorias (CIBERES), Instituto de Salud Carlos III (ISCIII), 08035 Barcelona, Spain; xmunoz@vhebron.net; 7 Pneumology Service, Hospital Vall d’Hebron Barcelona, 08035 Barcelona, Spain; 8 Department of Respiratory Medicine, University Hospital Complex of Santiago de Compostela, 15706 Santiago de Compostela, Spain; 9 Department of Medicine, Universidade de Santiago de Compostela, 15782 Santiago de Compostela, Spain

**Keywords:** proteomics, small extracellular vesicles, exosomes, immune cells, inflammatory diseases

## Abstract

Several studies have described the proteomic profile of different immune cell types, but only a few have also analysed the content of their delivered small extracellular vesicles (sEVs). The aim of the present study was to compare the protein signature of sEVs delivered from granulocytes (i.e., neutrophils and eosinophils) and CD4^+^ T cells (i.e., TH1, TH2, and TH17) to identify potential biomarkers of the inflammatory profile in chronic inflammatory diseases. Qualitative (DDA) and quantitative (DIA-SWATH) analyses of in vitro-produced sEVs revealed proteome variations depending on the cell source. The main differences were found between granulocyte- and TH cell-derived sEVs, with a higher abundance of antimicrobial proteins (e.g., LCN2, LTF, MPO) in granulocyte-derived sEVs and an enrichment of ribosomal proteins (RPL and RPS proteins) in TH-derived sEVs. Additionally, we found differentially abundant proteins between neutrophil and eosinophil sEVs (e.g., ILF2, LTF, LCN2) and between sEVs from different TH subsets (e.g., ISG15, ITGA4, ITGB2, or NAMPT). A “proof-of-concept” assay was also performed, with TH2 biomarkers ITGA4 and ITGB2 displaying a differential abundance in sEVs from T2^high^ and T2^low^ asthma patients. Thus, our findings highlight the potential use of these sEVs as a source of biomarkers for diseases where the different immune cell subsets studied participate, particularly chronic inflammatory pathologies such as asthma or chronic obstructive pulmonary disease (COPD).

## 1. Introduction

Extracellular vesicles (EVs) are lipid particles secreted into the extracellular space by all cell types, including immune cells [1]. Small extracellular vesicles (sEVs) are a heterogeneous group of nanosized (30–150 nm) vesicles which are generated through the endosomal route [1]. sEVs play a key role in cell-to-cell communication, as they carry a variety of macromolecules, including proteins (present on the surface and within the vesicle), nucleic acids (mRNA, miRNA, or DNA), lipids, and metabolites that may interact with surface receptors or be internalised by recipient cells. sEVs have the capacity to function as immune modulators, presenting antigens to T cells and, depending on their biomolecular composition, either promoting the activation or suppression of immune cells [2]. Furthermore, sEVs can be potentially used as a therapeutic approach to treat immune-related diseases [3].

The potential of sEV cargo to serve as biomarkers for the assessment of leukocyte activation and immune-related pathologies is contingent upon the cell of origin and its status. This is exemplified in conditions such as chronic lung diseases and autoimmune disorders, where there is an intense immune dysregulation and specific sEV-associated miRNAs have been reported as promising biomarkers. For instance, patients diagnosed with chronic obstructive pulmonary disease (COPD) display varying levels of specific sEV-associated miRNAs (e.g., miR-210, miR-144, miR-101, or miR-223) in comparison to healthy individuals [4]. In the same vein, our studies have shown differential levels of miR-21-5p, miR-126-3p, miR-146a-5p, and miR-215-5p in serum sEVs of asthma patients separated by phenotype or disease severity [5]. Similarly, several sEV miRNAs have been identified as biomarkers for autoimmune diseases, including miR-451a and miR-25-3p for rheumatoid arthritis (RA) and miR-21 and miR-155 for systemic lupus erythematosus (SLE) [6]. However, in contrast to the extensive literature regarding the differential abundance of different sEV-associated miRNAs in immune-based diseases, there are fewer studies where the sEV proteome is analysed. Some examples are fibulin-3 in COPD [7] or SAA1 in ankylosing spondylitis [8].

EVs in plasma or serum are primarily derived from platelets, erythrocytes, B cells, T cells, and other leucocytes, and their cargo (e.g., the miRNA profile) is deeply influenced by this cell origin and the activation status [9,10]. Thus, de Candia et al. have demonstrated that different lymphocyte subpopulations have specific miRNA signatures within the serum miRNome [11]. This indicates that the observed alterations in the circulating sEV cargo (e.g., miRNAs, proteins) between pathological and healthy states may reflect the shift in the predominant immune cell types involved in disease pathogenesis. Nevertheless, despite several cross-sectional studies on different diseases and the protein signatures of sEVs derived from plasma/serum samples [12,13], or studies evaluating the protein profiles of sEVs from diverse immune cell populations [14,15,16], the complexity of such analyses (e.g., difficult to isolate leukocyte subsets, minute amounts of sEVs produced, contamination with high abundance of plasma proteins) gives rise to the necessity of additional studies.

In the present work, we have performed a qualitative (DDA) and quantitative (DIA-SWATH) comparison of the proteomic profile of sEVs delivered by the main cell types involved in chronic inflammatory diseases: granulocytes (eosinophils and neutrophils) and CD4^+^ T-helper (TH) subsets (TH1, TH2, and TH17). Our results demonstrate a distinct proteome signature of sEVs depending on the cell type of origin, with the presence of potential cell lineage biomarkers. In addition, a preliminary proof-of-concept study on circulating sEVs from asthma patients shows that this panel of biomarkers may be useful in unravelling the underlying disease heterogeneity in several immune-related pathologies.

## 2. Results

### 2.1. Isolation of Human Eosinophils, Neutrophils, and CD4^+^ T Lymphocytes from Peripheral Blood and In Vitro Differentiation of TH1, TH2, and TH17 Subpopulations

**Figure 1:**
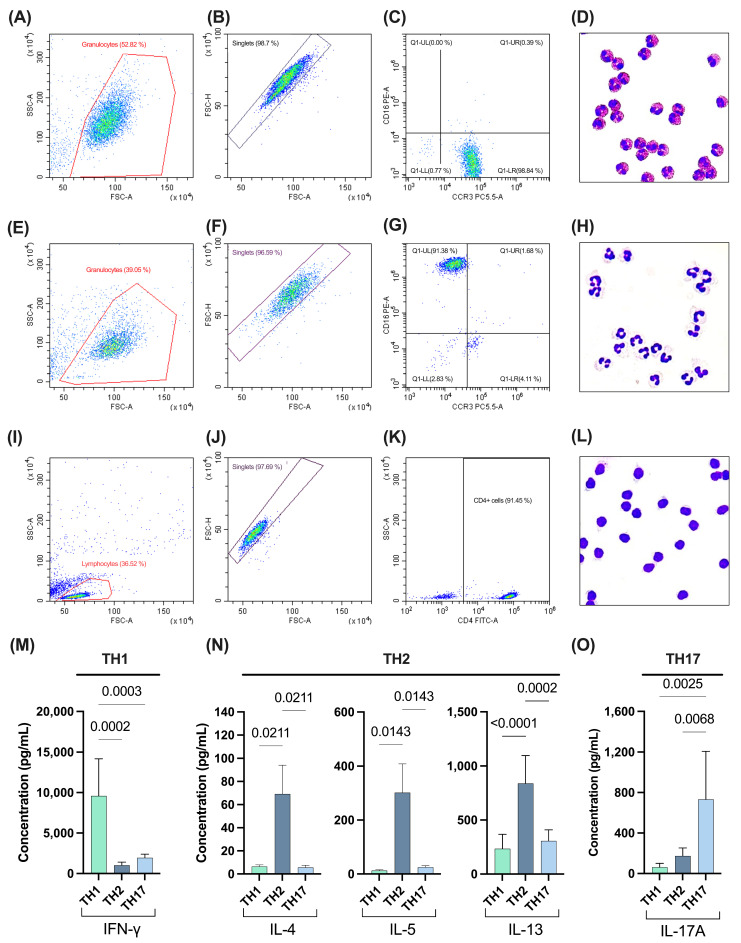
Characterisation of the studied leukocyte subpopulations. (**A**–**D**) Characterisation of eosinophils isolated from peripheral blood using flow cytometry (**A**–**C**) and cytospin with Kwik-Diff staining (**D**). (**E**–**H**) Characterisation of neutrophils isolated from peripheral blood using flow cytometry (**E**–**G**) and cytospin with Kwik-Diff staining (**H**). (**I**–**L**) Characterisation of T-helper (TH; CD4^+^) cells isolated from peripheral blood using flow cytometry (**I**–**K**) and cytospin with Kwik-Diff staining (**L**). A representative picture for each cell subpopulation is depicted. (**M**–**O**) Levels of prototypical TH1 (IFN-γ; (**M**)), TH2 (IL-4, IL-5, and IL-13; (**N**)), and TH17 (IL-17A; (**O**)) cytokines produced by TH cells upon PMA + ionomycin activation measured by multiplex ELISA (N = 3).

In the present study, we have performed a qualitative (DDA) and quantitative (DIA-SWATH) comparison of the proteomic profile of sEVs delivered by the main cell types involved in chronic inflammatory diseases: granulocytes (eosinophils and neutrophils) and CD4^+^ T-helper subsets. First, eosinophils and neutrophils were isolated from peripheral blood with high purity (>90% of CCR3^+^CD16^−^ and CCR3^−^CD16^+^; Figure 1A–H). Similar results were obtained when isolating CD4^+^ T cells (TH cells) from buffy coats (>90% of CD4^+^ T cells; Figure 1I–L). Afterwards, TH cells were expanded (7 days), activated (anti-CD3/CD28), and differentiated in vitro to the major TH subsets (TH1, TH2, and TH17). The efficacy of the differentiation protocol was assessed with a multiplex Luminex immunoassay (Figure 1Μ–O). As expected, upon activation with Phorbol 12-myristate 13-acetate (PMA) + ionomycin, TH1 cells secreted high levels of IFN-γ, the major type-1 cytokine (Figure 1M), TH2 lymphocytes released high quantities of prototypical TH2 cytokines (i.e., IL-4, IL-5, and IL-13; Figure 1N), and TH17 cells produced high amounts of IL-17A (Figure 1O). Altogether, our results indicated a high level of purity and/or differentiation in all the leukocyte populations and subsets under study prior to in vitro activation and sEV isolation.

**Figure 2:**
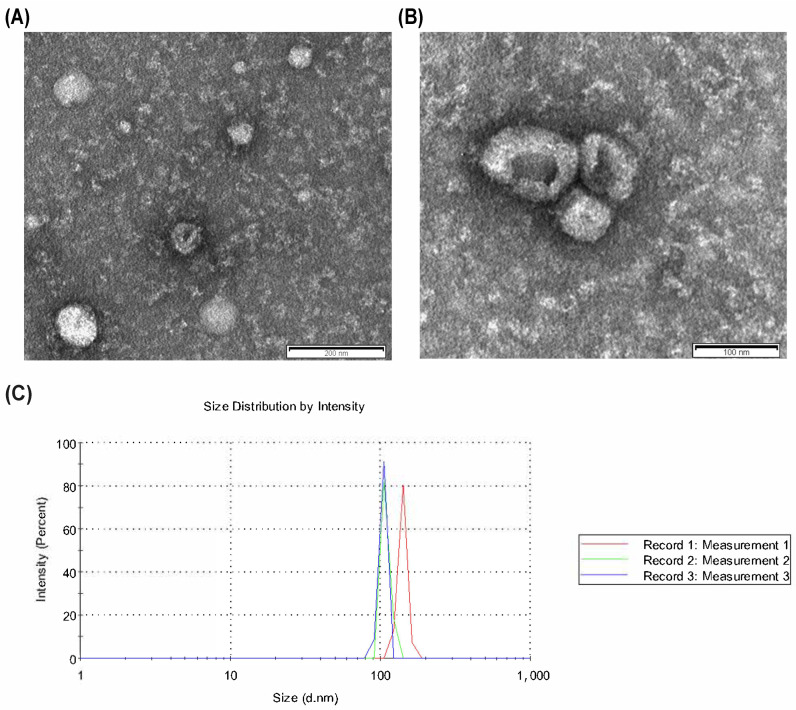
Characterisation of small extracellular vesicles (sEVs). (**A**,**B**) Representative images from transmission electron microscopy (TEM). The scale bars of images are 200 nm (**A**) and 100 nm (**B**). The acquisition was at an accelerating voltage of 40–100 kV. (**C**) Dynamic light scattering (DLS) measurement of the size of nanovesicles indicating peaks from 90 to 150 nm (i.e., sEV/exosome size). Three representative measurements are depicted.

As sEV release is triggered by increasing intracellular calcium concentration and PKC activation, we have stimulated in vitro sEV production from the different leukocyte populations (i.e., neutrophils, eosinophils, TH1, TH2, TH17) using PMA + ionomycin. sEVs isolated from a conditioned medium by ultracentrifugation had sizes in the sEV range (purity grade was high according to dynamic light scattering (DLS) curves; indicating a 50–150 nm diameter) and a typical bilayer membrane and cap-shaped vesicle structure (transmission electron microscopy, TEM) (Figure 2). To verify these data, western blot analyses were also performed on sEV lysates to detect some members of the tetraspanin family (CD9, CD81, CD63), which are highly enriched in these vesicles. Unfortunately, we could not detect these proteins in sEV lysates from the different leukocyte subsets (eosinophils, neutrophils, and TH subsets). As CD9 and CD63 are present in sEVs from T cells [14,17], neutrophils [18], and eosinophils [19], our negative results perhaps stem from the limited amount of sEVs purified (70–80 μg/1 × 10^6^ cells) and sEV protein loaded per lane (<10 μg/well) compared to previous works [17]. Furthermore, the use of reducing conditions in our assays or the higher abundance of CD63 on the membrane of monocytes compared to neutrophils, eosinophils, lymphocytes, and especially CD4^+^ T cells (see flow cytometry data in Supplementary Figure S1) could also play a role. In conclusion, despite some constraints, our experimental results supported that our sEVs could be used for subsequent proteomic experiments.

### 2.2. Qualitative and Comparative Analyses of sEV Proteins from Different Leukocyte Subpopulations

**Figure 3:**
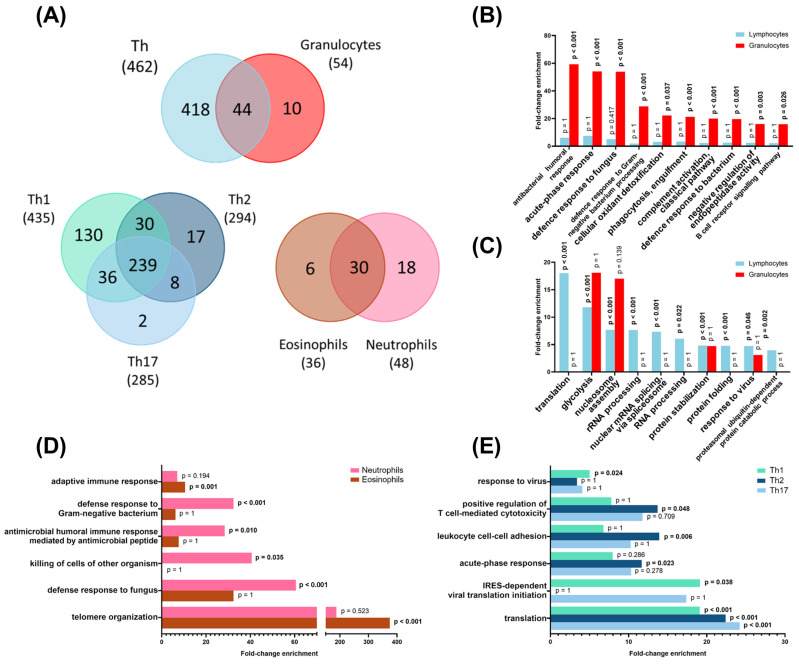
Qualitative proteomic analyses of sEVs from different leukocyte subpopulations. (**A**) Venn diagrams of identified proteins in CD4^+^ T lymphocytes (TH cells) vs. granulocytes, eosinophils vs. neutrophils, and TH1 vs. TH2 vs. TH17. X-axes represent fold-change enrichment of the biological process (BP) over the whole human proteome. The ten most enriched BP in lymphocytes (CD4^+^ T cells) (**B**) and granulocytes (**C**) are depicted. The six processes with bigger changes between neutrophils and eosinophils (**D**) or between the different TH subpopulations (**E**) are shown.

Two types of proteomic analyses were then performed in sEVs secreted by the isolated leukocyte subpopulations: (1) qualitative analysis by LC-MS/MS (DDA) and (2) label-free quantification by DIA (SWATH-MS/MS). Regarding the qualitative analysis (DDA) of sEVs, the number of isolated proteins from each group of sEVs is depicted in Figure 3A (Supplementary Table S1). As expected, most of the identified proteins (i.e., 88% of the proteins from lymphocyte-derived sEVs and 76% of the proteins from granulocyte-derived sEVs were present in the ExoCarta database (Supplementary Figure S2). Among the identified proteins, 44 proteins were shared between TH cell- and granulocyte-derived sEVs, 239 were common to all TH-delivered sEVs independently of the cell subset, and 30 proteins were common to all granulocytes-derived sEVs (Figure 3A). Interestingly, a functional enrichment analysis of the identified proteins highlighted a clear difference between sEVs from granulocytes and CD4^+^ T lymphocytes (Figure 3B,C). Thus, granulocyte sEVs are enriched in proteins with innate immune-related functions, such as acute-phase response, and response to bacteria, fungi, phagocytosis, or complement activation (Figure 3B). Broadly speaking, proteins upregulated in sEVs from TH lymphocytes were implicated in translation or transcription, but the GO term “response to virus” was also enriched in TH sEVs vs. granulocyte sEVs (Figure 3C).

By comparing the identified sEV-associated proteins within granulocyte subpopulations, we found an enrichment in antimicrobial response pathways in neutrophil-derived sEVs, whereas the upregulation of the telomere organization process was highlighted in sEVs released by eosinophils (Figure 3D). On the other hand, the three studied TH subsets also showed an enrichment in certain specific patterns of sEV-associated proteins in terms of GO-BP categories. Thus, viral response pathways were enriched in proteins from TH1 sEVs, whereas cell-to-cell adhesion processes were overrepresented amongst sEV proteins from TH2 cells (Figure 3E).

### 2.3. Quantitative Analyses of sEV Proteins from Different Leukocyte Subpopulations

To obtain a clear picture of the sEV proteins from different leukocyte subpopulations, we carried out a quantitative analysis using DIA (SWATH-MS/MS) label-free proteomics. Thus, we wanted to determine the enriched or reduced content in sEV proteins across the following comparisons: granulocytes vs. CD4^+^ T cells, eosinophils vs. neutrophils, and within the different TH subsets (TH1, TH2, and TH17).

A total of 314 proteins were identified in SWATH-MS/MS analyses (Supplementary Table S2). Six of these were considered contaminants and removed from further analysis (keratins KRT1, KRT2, KRT9, KRT10, and KRT14; POLG, HVC protein). In addition, we have noticed the presence of some highly abundant serum proteins (N = 24) in our samples, and the co-isolation of these proteins was not the same in the different sample groups (Supplementary Figure S3). Therefore, to avoid bias in differential abundance analyses, we additionally removed these highly abundant serum proteins from the analysis before normalisation by the total sum of the remaining (sEV) proteins (N = 284; Supplementary Table S3).

**Figure 4:**
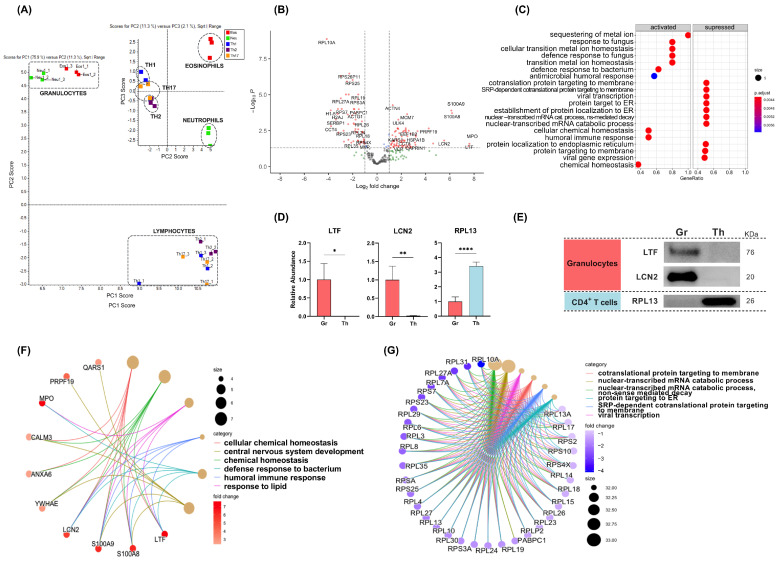
Quantitative proteomic analysis of sEV proteins from granulocytes compared to CD4^+^ T cells. (**A**) Principal component analysis (PCA) of sEV protein expression in the study subpopulations. Square root range transformed data for quantitative comparison of all samples (N = 3 for each cell subpopulation). Principal component 1 (PC1) and PC2 allow for separation of granulocytes vs. lymphocytes. PC2 vs. PC3 allows for separation of the other cell subpopulations. (**B**) Volcano plot showing differentially expressed proteins in sEVs from granulocytes vs. CD4^+^ T cells. (**C**) GO term enrichment (biological processes; BP) dot plot with the most up- and downregulated proteins in granulocytes vs. CD4^+^ T cells. (**D**) Bar graphs for the sEV relative abundance (normalised to granulocytes) of LTF, LCN2, and RPL13 in granulocytes (Gr) and CD4^+^ T cells (TH) measured by LC-MS/MS. (**E**) Representative western blot showing LTF, LCN2, and RPL13 protein abundance in Gr- vs. TH-derived sEVs (10 µg). (**F**,**G**) Category netplot of the 6 most upregulated pathways (GSEA) in granulocytes (**F**) and CD4^+^ T cells (**G**). * *p* < 0.05; ** *p* < 0.01; **** *p* < 0.0001.

A principal component analysis (PCA) for quantitative comparison of sEV proteins from all cell subpopulations is shown in Figure 4A. The PCA analysis revealed a clear separation of sEVs according to the cell subpopulation from which they were derived. Thus, a PC1 vs. PC2 score showed an important difference between sEVs from CD4^+^ T lymphocytes and those released by granulocytes. Furthermore, a PC2 vs. PC3 score allowed for the distinction of eosinophil sEVs from neutrophil sEVs and, although with less clear differences, between sEVs produced by the different TH-cell subsets.

### 2.4. Quantitative Changes in the Proteome of sEV Differentiate Vesicles from Granulocytes and CD4^+^ T Lymphocytes

Out of the 284 proteins, 69 proteins were upregulated in granulocyte sEVs and 52 in CD4^+^ T-cell sEVs (FC > 1; *p* < 0.05) (Figure 4B; Supplementary Table S4). Gene set enrichment analyses (GSEA) for GO-BP indicated that proteins mainly implicated in innate immune response processes (e.g., response to fungi or bacteria) were enriched in granulocyte-derived sEVs (Figure 3C), whereas most of the differentially abundant proteins associated with CD4^+^ T-cell sEVs were involved in protein metabolism/translational processes (Figure 4C). Indeed, 27 out of the 52 proteins found enriched in TH-derived sEVs were ribosomal proteins, belonging to either large (RPLs; N = 21) or small (RPSs; N = 6) subunits (Supplementary Table S4). Proteomic changes in LTF and LCN2 as specific granulocyte sEV proteins, and RPL13 as a representative RPL protein enriched in CD4^+^ T-cell sEVs (Figure 4D), were further validated by western blot (Figure 4E).

The six most upregulated processes, based on the normalised enrichment score (NES), associated with proteins found enriched in granulocyte sEVs were “GO:0055082: cellular chemical homeostasis” (NES = 2.041; *p* < 0.001), “GO:0048878: chemical homeostasis” (NES = 2.041; *p* < 0.001), “GO:0002237: response to lipid” (NES = 1.963; *p* < 0.001), “GO:0006959: humoral immune response” (NES = 1.961; *p* < 0.001), “GO:0032496: defense response to bacterium” (NES = 1.957; *p* < 0.001), and “GO:0007417:central nervous system development” (NES = 1.956; *p* < 0.001) (Figure 4F). Interestingly, overrepresentation analyses for Reactome pathways of the most enriched proteins in granulocyte-derived sEVs (FC > 2; *p* < 0.05) yielded similar results (Supplementary Table S5), with the most upregulated pathways being “HSA-6799990: Metal sequestration by antimicrobial proteins” (Strength = 2.52; FDR = 2.29 × 10^−6^), which includes the following four proteins: LTF (FC = 7.6; *p* = 0.014), S100A8 (FC = 6.2; *p* < 0.001), S100A9 (FC = 6.1; *p* < 0.001), and LCN2 (FC = 5.5; *p* = 0.006); and “HSA-6798695: Neutrophil degranulation” (Strength = 1.38; FDR = 4.13 × 10^−6^), including the four above-mentioned proteins, MPO (FC = 7.5; *p* = 0.025), and proteins like PIGR (FC = 3.1; *p* = 0.010), or HSPA1B (FC = 3.2; *p* = 0.003); all of them related to neutrophils (Supplementary Figure S4A; Supplementary Table S5).

On the other hand, GSEA analyses resulted in “GO:0000956: nuclear-transcribed mRNA catabolic process” (NES = −2.389; *p* < 0.001), “GO:0000184: nuclear-transcribed mRNA catabolic process, nonsense-mediated decay” (NES = −2.389; *p* < 0.001), “GO:0019083: viral transcription” (NES = −2.334; *p* < 0.001), “GO:0006613: cotranslational protein targeting to membrane” (NES = −2.337; *p* < 0.001), “GO:0006614: SRP-dependent cotranslational protein targeting to membrane” (NES = −2.337; *p* < 0.001), and “GO:0045047: protein targeting to ER” (NES = −2.337; *p* < 0.001) as the most upregulated processes in CD4^+^ T cells compared to granulocytes (Figure 4G). Similarly, 23 proteins were found to be highly enriched (FC > 2; *p* < 0.05) in CD4^+^ T cell-derived sEVs, with 13 of them belonging to the KEGG pathway “hsa03010:Ribosome (strength = 1.95; FDR = 1.52 × 10^−20^)” or other Reactome pathways related to translation that were overrepresented in comparison to the entire human proteome (see Supplementary Figure S4B and Supplementary Table S5).

### 2.5. The Proteome of sEVs Released by Eosinophils Differs from That of sEVs Produced by Neutrophils

**Figure 5:**
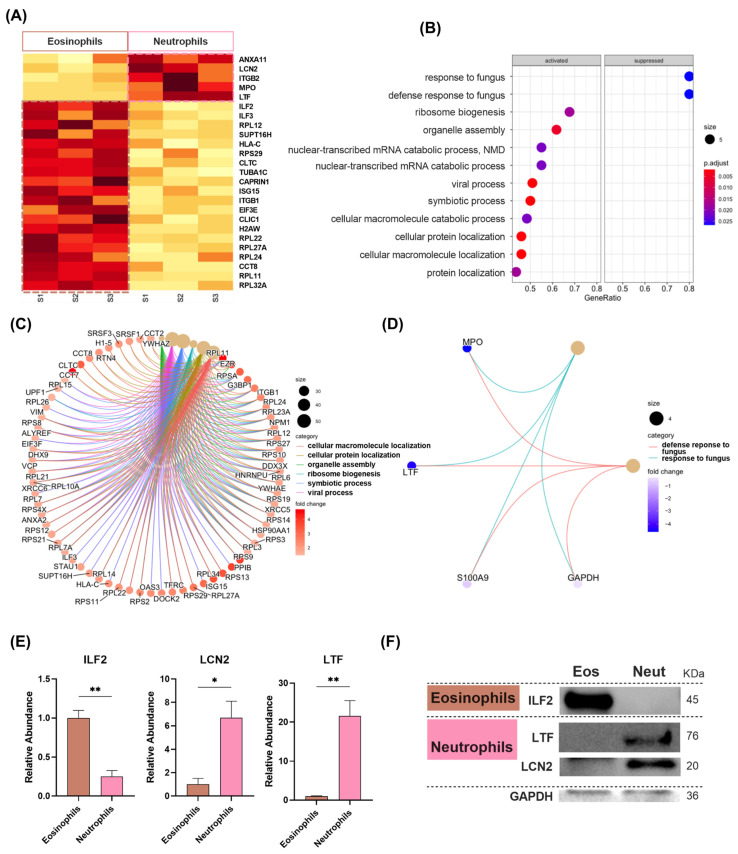
Quantitative proteomic analysis of sEV proteins from eosinophils compared to neutrophils. (**A**) Heatmap for the 25 more differentially expressed proteins with fold-change FC > 1 between eosinophil- and neutrophil-delivered sEVs. (**B**) GO term enrichment (biological processes; BP) dot plot with the most up- and downregulated proteins in sEVs from eosinophils vs. neutrophils. (**C**,**D**) Category netplot of the 6 most upregulated pathways (GSEA) in eosinophil- (**C**) and neutrophil-derived exosomes (**D**). (**E**) Bar graphs for the sEV relative abundance (normalised to eosinophils) of ILF2, LCN2, and LTF in eosinophils vs. neutrophils measured by LC-MS/MS. (**F**) Representative western blot showing ILF2, LCN2, and LTF protein abundance in sEVs from eosinophils (Eos) vs. neutrophils (Neut) (2 µg). * *p* < 0.05; ** *p* < 0.01.

Next, we analysed the differences in sEV proteomes from eosinophils and neutrophils. We found 47 proteins upregulated in eosinophil-derived sEVs (FC > 1; *p* < 0.05) and five proteins upregulated in neutrophil-delivered sEVs out of the 284 identified proteins (Figure 5A; Supplementary Table S6).

Interestingly, within the most upregulated proteins in eosinophil sEVs (FC > 2, *p* < 0.05), we found an overrepresentation in KEGG of “hsa03010:Ribosome (Strength = 1.75; FDR < 0.001)”, which included six ribosomal subunits (RPL11, RPL22, RPL23A, RPL24, RPL27A, RPS29), as well as “hsa04145:Phagosome (Strength = 1.42; FDR < 0.033)”, where ITGB1, TUBA1C, and HLA-C were present (Supplementary Table S7). GSEA enrichment analyses of proteins upregulated in eosinophil-derived sEVs yielded similar results (Figure 5B), as well as the Reactome pathways overrepresented (Supplementary Table S7). The six most upregulated gene sets for GO-BP, with the core genes identified in our samples, are depicted in Figure 5C.

On the other hand, only two gene sets (GSEA analyses) were significantly enriched in sEVs produced by neutrophils: “GO:0009620: response to fungus” and “GO:0050832: defense response to fungus”, both with NES = 2.16 (*p* < 0.001) and made of the typical neutrophil granule proteins MPO (FC = 4.60; *p* = 0.034), LTF (FC = 4.43; *p* = 0.006), and S100A9 (FC = 0.78; *p* = 0.152) (Figure 5B,D). An overrepresentation analysis of highly upregulated proteins (FC > 2; *p* < 0.05) in neutrophil-derived sEVs identified two enriched Reactome pathways: “metal sequestration by antimicrobial proteins” (Strength = 3.22; FDR < 0.001), and “neutrophil degranulation” (strength = 1.62; FDR < 0.001) (Supplementary Table S7). The upregulation of ILF2 on eosinophil-derived sEVs, and LTF and LCN2 on neutrophil-derived sEVs found in LC-MS/MS (Figure 5E), was further validated by western blot (Figure 5F).

### 2.6. Quantitative Proteomic Analysis Revealed a Distinct Protein Pattern of sEV between TH1 and TH2 Lymphocyte Subpopulations

**Figure 6:**
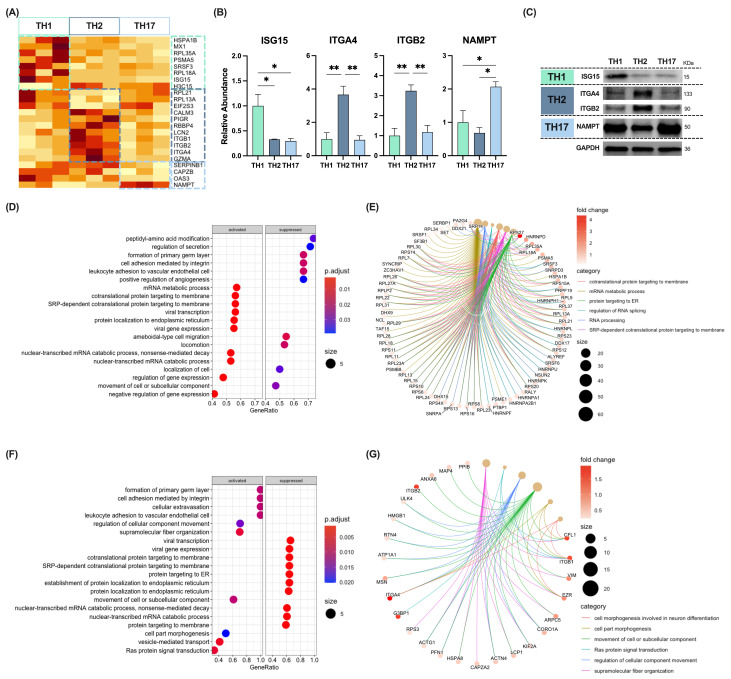
Quantitative proteomic analysis of sEV proteins from different CD4^+^ T-cell subpopulations. (**A**) Heatmap of differentially expressed proteins between TH1, TH2, and TH17 cells. (**B**) Bar graphs for the sEV relative abundance (normalized to TH1) of ISG15, ITGA4, ITGB2, and NAMPT in CD4^+^ T-cell subpopulations measured by LC-MS/MS. (**C**) Representative western blot showing ISG15, ITGA4, ITGB2, and NAMPT protein abundance in TH1, TH2, and TH17-delivered sEVs (10 µg). (**D**) GO term enrichment (biological processes) dot plot with the most up- and downregulated proteins in TH1 sEVs compared to the other CD4^+^ T cells. (**E**) Category netplot of the 6 most up-regulated pathways in TH1 cell-derived sEVs. (**F**) GO term enrichment (biological processes) dot plot with the most up- and downregulated proteins in TH2 sEVs compared to the other CD4^+^ T cells. (**G**) Category netplot of the 6 most upregulated pathways in TH2 cell-derived sEVs. * *p* < 0.05; ** *p* < 0.01.

We also performed a differential proteome analysis in sEVs released in vitro by three of the major CD4^+^ TH subsets: TH1, TH2, and TH17. The results of our study demonstrated that 23 proteins exhibited differential abundance (FC > 1; *p* < 0.05) between sEVs derived from the various TH subpopulations (Figure 6A; Supplementary Table S8). Of the proteins examined, only NAMPT and three others (OAS3, CAPZB, and SERPINB1) were found to be up- and downregulated, respectively, in sEVs derived from TH17 cells in comparison to those derived from other CD4^+^ T cells (Figure 6A). Conversely, our findings indicated that eight and twelve proteins displayed a differential abundance in sEVs released by TH1 and TH2 lymphocytes, respectively, in comparison to other CD4^+^ T-cell subpopulations (Figure 6A). The upregulation of ISG15 on TH1-, ITGA4 and ITGB2 on TH2-, and NAMPT on TH17-derived sEVs found by LC-MS/MS (Figure 6B) was further validated by western blot (Figure 6C).

GSEA analyses yielded no enrichments in GO-BP terms for TH17 cells. However, different processes were either up- or downregulated amongst the proteins associated with sEVs from TH1 cells (Figure 6D,E; Supplementary Figure S5A). Upregulated processes in the proteome of TH1 cell-derived sEVs were mainly connected to protein localisation or RNA processing (e.g., translation, regulation of RNA splicing) and included different ribosomal proteins (RPL, RPS, and hnRNP proteins) (Figure 6E). Thus, the six most upregulated processes in the proteome of TH1 cell-derived sEVs vs. the proteome of other CD4^+^ T subsets included “GO:0016071: mRNA metabolic process” (NES = 2.264; *p* = 0.002), “GO:0006396: RNA processing” (NES = 2.158; *p* = 0.002), “GO:0043484: regulation of RNA splicing” (NES = 2.069; *p* = 0.009), “GO:0006613: cotranslational protein targeting to membrane” (NES = 2.059; *p* = 0.002), “GO:0006614: SRP-dependent cotranslational protein targeting to membrane” (NES = 2.059; *p* = 0.002), and “GO:0045047: protein targeting to ER” (NES = 2.059; *p* = 0.002) (Figure 6E). Overrepresentation analyses of upregulated proteins yielded six Reactome pathways enriched, highlighting “HSA-5663205: Infectious disease” (strength = 1.19; FDR = 0.003), which included the proteins RPL18A, PSMA5, HSPA1B, RPL35A, and ISG15 (Supplementary Table S9).

On the other hand, the proteome of TH2 cell-derived sEVs also displayed several up- and downregulated processes (Figure 6F,G; Supplementary Figure S5B). Particularly, BP terms enriched in the proteome of TH2 cell-derived sEVs included proteins that participate in cell adhesion, such as integrins (e.g., ITGA4, ITGB1, and ITGB2). Thus, the six most upregulated processes were “GO:0097435: supramolecular fiber organization” (NES = 2.043; *p* = 0.005), “GO:0.0007265: Ras protein signal transduction” (NES = 1.988; *p* = 0.004), “GO:0051270: regulation of cellular component movement” (NES = 1.946; *p* = 0.016), “GO:0006928: movement of cell or subcellular component” (NES = 1.900; *p* = 0.010), “GO:0032990: cell part morphogenesis” (NES = 1.883; *p* = 0.020), and “GO:0048667: cell morphogenesis involved in neuron differentiation” (NES = 1.883; *p* = 0.020) (Figure 6G). In addition, several pathways (both Reactome and KEGG) were significantly overrepresented within proteins upregulated in TH2 cell-derived sEVs (Supplementary Table S9); amongst them were pathways related to T2-type inflammation, such as “HSA-6785807: Interleukin-4 and Interleukin-13 signaling” (Strength = 1.79, FDR = 0.008), or related to transendothelial migration, such as “HSA-216083: Integrin cell surface interactions” (Strength = 1.89, FDR = 0.008), or “HSA-202733: Cell surface interactions at the vascular wall” (Strength = 1.67, FDR = 0.013).

### 2.7. Levels of Biomarkers Associated with TH2-Derived Small Extracellular Vesicles (ITGA4 and ITGB2) Are Increased in T2^high^ Compared to T2^low^ Asthma Donors

**Figure 7:**
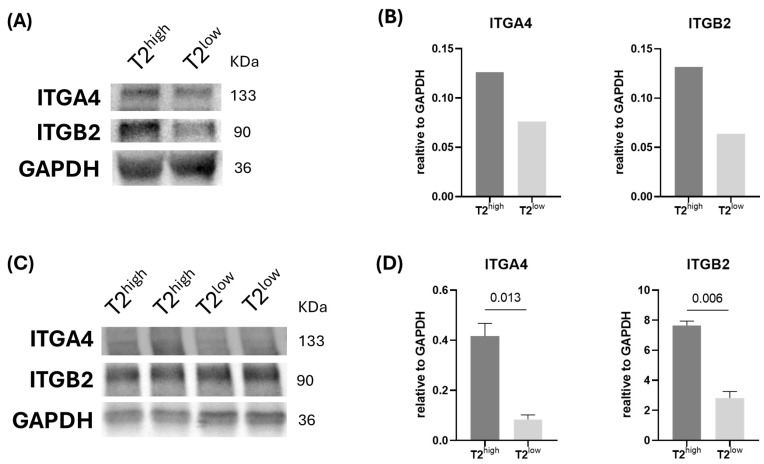
TH2 biomarkers ITGB2 and ITGB4 are enhanced in sEVs purified from serum samples from T2^high^ compared to T2^low^ asthma patients. sEVs were isolated from serum samples from T2^high^ and T2^low^ asthma donors with (**A**,**B**) ultracentrifugation (1 pool/condition) or (**C**,**D**) “Total Exosome Isolation Reagent (from serum)” (Invitrogen^TM^) (2 pools/condition). Western blot analysis of ITGA4, ITGB2, and GADPH (**A**,**C**) and normalised levels of ITGA4 and ITGB2 relative to the GAPDH abundance (**B**,**D**). In (**D**) a *t*-test was performed.

To perform a preliminary evaluation of the clinical applicability of our set of protein markers, we carried out a limited proof-of-concept test with two biomarkers associated with TH2-derived sEVs (ITGA4 and ITGB2) and took advantage of a previous collection of serum samples from T2^high^ and T2^low^ asthma patients [5,20], two molecular phenotypes where TH2 cells play a differential role. Two sEV isolation methods were employed for comparative purposes: (a) the previously utilised ultracentrifugation approach and (b) a rapid commercial method (“total exosome isolation reagent” from Invitrogen) that is more readily adaptable for clinical use. To obtain enough protein from sEVs for these assays, equal amounts of serum samples from randomly selected patients were pooled according to their condition: one pool for UC (N = 4) and two pools for TEI (N = 3 each). As hypothesised, western blot assays revealed the presence of both biomarkers in lysates of sEVs from serum purified by ultracentrifugation (Figure 7A, western blot analysis of ITGA4, ITGB2, and GADPH; B, normalised signal density by GAPDH), or TEI (Figure 7B, western blot analysis of ITGA4, ITGB2, and GADPH; Figure 7C, signal density of ITGA4 and ITGB2 normalised by GAPDH) with bands of higher intensity for T2^high^ asthma compared to T2^low^ asthma. Therefore, even though these are only preliminary data and there is scope for enhancement, these findings corroborate our initial hypothesis and lend support to the applicability of this panel of sEV-associated biomarkers from neutrophils, eosinophils, and TH1/TH2/TH17 cells for the characterisation of chronic inflammatory responses in a range of diseases.

## 3. Discussion

The molecular cargo of sEVs is variable and highly related to the cellular origin. In the present study, we have isolated different subpopulations of granulocytes (eosinophils and neutrophils) and CD4^+^ T lymphocytes (TH1, TH2, TH17), which are markedly important for the definition of chronic inflammatory diseases. Both qualitative (DDA) and quantitative (DIA-SWATH) proteomic analyses evidenced a clear separation of sEVs according to the cell source. Generally, granulocyte-derived sEV proteins are related to innate immune system processes and have effector functions, whereas sEV proteins from TH cells are enriched in ribosomal proteins involved in translation processes. In addition, comparative analyses of granulocyte-delivered sEVs reveal that proteins involved in anti-microbial responses (e.g., LTF, MPO, LCN2, or calgranulins) are enriched in sEVs derived from neutrophils relative to those from eosinophils. Furthermore, sEVs from the different TH-lymphocyte subsets also differ in their protein cargo, specially TH1 and TH2 cell-derived sEVs. Thus, TH1 cell-derived sEVs carry proteins that play a role in the immune system’s response to infectious diseases, particularly those of viral origin, whereas sEV proteins from TH2 cells display adhesion functions. This is, to the best of our knowledge, the first study to compare the proteome of sEVs derived from so many immune cell subpopulations at once with a key role in the definition of chronic inflammatory diseases.

Both eosinophils and neutrophils are key modulators in chronic inflammatory diseases, including several allergic and chronic respiratory disorders [21,22]. Indeed, the levels of these granulocytes either in peripheral blood or sputum are currently used for the definition of specific asthma/COPD phenotypes and severity levels [21,23,24,25]. Moreover, many of the novel treatment strategies in allergic disorders (e.g., atopic dermatitis) and chronic respiratory diseases (severe eosinophilic asthma, EGPA, eosinophilic COPD, HES) specifically target eosinophils (i.e., mepolizumab/anti-IL-5 mAb or benralizumab/anti-IL-5R mAb) [26]. Therefore, to find lineage markers associated with sEVs from eosinophils and neutrophils is of high relevance. Cañas et al. performed in 2017 the first known proteome (RP-LC-MS/MS) analysis of sEVs released by human eosinophils, detecting proteins implicated in asthma pathogenesis (e.g., ECP, EPO, MBP, periostin) and linked to migration, adhesion, cell signalling, redox, or inflammation [27]. In line with these results, our study reports the presence of adhesion molecules (e.g., ITGB1) in the proteome of eosinophil sEVs, but also chromatin-interacting (e.g., ILF2, ILF3), ubiquitin-like (e.g., ISG15, also found in TH1-derived sEVs, see below), or mRNA-binding proteins (e.g., CAPRIN1).

On the other hand, Shao et al. reported in 2019 the presence of certain proteins (LTF, MPO, S100A8/S100A9, ELANE, SERPINB1, and MMP9) by LC-MS/MS analysis of neutrophil exosome preparations [28], and a similar proteomic profile (e.g., ELANE, MPO, CTSG, AZU1, DEFA1) was described by Shopova et al. in 2020 in extracellular vesicles from *Aspergillus fumigatus*-triggered PMN granulocytes [29]. By comparing the sEV proteome of eosinophils and neutrophils by label-free LC-MS/MS, we have also found an upregulation of proteins with anti-microbial functions in neutrophil-derived sEVs, particularly LTF, MPO, LCN2, and calgranulins (S100A8/S100A9). LTF anti-microbial activity is well documented. On the one hand, LTF has a bacteriostatic function though iron sequestering [30]; this function is also shared with neutrophil-derived LCN2 [31]. On the other hand, LTF also binds to bacterial LPS, and induces cell breakdown through peroxide formation [30]. MPO is a peroxidase enzyme that produces cytotoxic mediators (i.e., hypochlorous or hypobromous acid) from hydrogen peroxide (H_2_O_2_), which are used by neutrophils to kill pathogens [32]. Some of those neutrophil proteins are also present in larger (200–500 nm) primary (e.g., MPO) and secondary (e.g., LTF) granules released by these cells [16,33], which are linked to neutrophilic inflammation in various pathological conditions. For example, LTF, MPO, LCN2, and S100A9 are markers of neutrophil degranulation previously associated with bacterial pneumonia [34]. LTF and LCN2 were also induced during a septic shock [35], and MPO, LCN2, and LTF were used to distinguish between latent and active tuberculosis [36]. LTF is induced in children upon influenza A infection to prevent secondary bacterial infections [37]. Furthermore, LTF and LCN2 also belong to the core genes associated with clinical severity in influenza infections [38], while LTF and S100A9 were associated with a higher mortality in patients with COVID-19 disease [39]. In addition, COPD, a disease with a big neutrophilic component, is characterised by high levels of autoantibodies against LTF [33].

On the other hand, the study of neutrophil-associated sEV proteins (e.g., LTF) may also be relevant in allergic disorders. In this regard, several authors have demonstrated the potential protective or anti-inflammatory effect of LTF in allergy and asthma [40,41,42]. Hence, LTF polarises immune response towards a TH1 over TH2 response [43], and this protein is one of the most downregulated genes in asthma vs. healthy individuals [40,42]. Indeed, Fernández-Delgado et al. [41] have evidenced that neutrophils from allergic asthma donors release LTF in response to allergen challenges in vitro. This protein also reduces AHR, lung inflammation, and TH2 immune responses in different mouse models of airway allergy [44,45].

When comparing the sEV proteome between granulocytes and CD4^+^ T lymphocytes, we found a clear upregulation of proteins involved in macromolecule biosynthesis (i.e., ribosomal proteins/RPs) in sEVs from CD4^+^ T lymphocytes. A dominant signature of genes encoding for ribosomal proteins in lymphocytes vs. granulocytes was previously validated in different microarray-based analyses and different gene expression studies [46,47]. The presence of these translational regulators (i.e., RPs) may be required for compacting nucleic acid structures into sEV lumen. However, different studies also suggest that RPs delivered by sEVs might have an impact on recipient cells by regulating both protein production and cell phenotype [48]. Other studies highlighted the importance of sEVs from T cells as a form of intercellular communication. For example, activated CD3^+^ T cell-derived sEVs are able to induce proliferation of autologous resting CD3^+^ T cells [49]. Azoulay-Alfaguter I et al. have evidenced an upregulation of proteins from the RAS signalling pathway in sEVs after T-cell activation, which induces ERK phosphorylation in sEV-recipient cells [15].

sEV components can also be used as biomarkers of activation and functional status of the originating cells [14,50]. For example, serum miRNAs were previously described as biomarkers of lymphocyte activation (e.g., miR-150) or differentiation status [14,50]. Indeed, we have found that sEV miRNAs associated with different TH-cell subsets are modulated with the endotype and severity of asthma [5]. In addition, Zebrowska A et al. have shown that the proteomic and metabolomic profile of CD3^+^ sEVs isolated from plasma reflects the general activation and functional status of T cells in the donor [14]. As described above for eosinophils and neutrophils, CD4^+^ T cells are also key for definition of specific asthma (i.e., T2^high^ vs. T2^low^ asthma) and COPD (e.g., eosinophilic COPD) endotypes [51,52]. Moreover, TH1, TH2, and TH17 are the major CD4^+^ TH subsets involved in other immunological disorders [53]. Although there are some works comparing the proteome of different immune cell populations [54,55], none has studied the protein signatures of sEVs. Thus, we studied the sEV proteome of TH subsets after in vitro activation and differentiation. We found a clear separation between TH1 and TH2 cell-derived sEVs from a proteomic point of view.

Previous studies have suggested that anti-viral molecules can be transferred from infected cells in the sEV cargo [56]. Now, we describe that both eosinophils and TH1 cells release sEVs enriched in proteins with anti-viral functions. Furthermore, the upregulation of ISG15 on TH1 cell-derived sEVs was validated using a western blot. Strikingly, the ISG15 gene was recently found upregulated in whole blood from SLE, a typical T1-T17 autoimmune disease, and its levels decrease after treatment [57]. Moreover, the ISG15 gene was also increased in blood samples from patients infected with RSV vs. healthy controls [58]. ISG15 is a type I IFN-induced protein with a central role in host anti-viral immunity. On the one hand, mature ISG15 is post-translationally conjugated to cellular (host) and viral proteins in a process called ISGylation, resulting in the disruption of viral replication. On the other hand, and in line with our results, unconjugated ISG15 can also be released in sEVs [59]. Once released, ISG15 acts as a cytokine and mediates the chemotaxis of neutrophils, or the activation of NK and T cells to produce IFN-γ [59] through the activation of the LFA-1 (CD11a/CD18; αLβ2 integrin) receptor [60]. Interestingly, ISG15 also regulates disease tolerance after the infection by activating the repair response of the respiratory epithelium, potentially through the reorganization of the cytoskeleton of actin [61].

We have evidenced an increase in adhesion molecules in TH2 cell-derived sEVs, including several integrins (e.g., ITGA4, ITGB1, and ITGB2). One of these integrins, ITGA4 (CD49d), could have a key role in allergic conditions, where T2-type inflammation is characteristic. Indeed, the expression of CD49d increases under allergic stimulation [62], and the presence of a discrete memory subpopulation of TH2 cells with high levels of CD49d (TH2A) was described in allergic individuals [62]. ITGA4 was also related to allergic rhinitis associated with obesity, potentially due to its relevance on the leptin–osteopontin interaction in TH2 cells [63]. Strikingly, treatment with anti-ITGA4 antibodies on obese-ovalbumin (OVA) mice reduced the nasal inflammation [63], highlighting the relevance of our results from a therapeutic point of view. ITGA4 forms a dimer with ITGB1 (CD29; also upregulated in TH2-derived sEVs), called VLA-4. VLA-4 is the receptor for fibronectin and VCAM-1, and this interaction induces the leukocyte transmigration across the endothelial membrane towards the inflamed tissues [64]. Apart from the role of these integrins in cell adhesion, ITGA4 and ITGB1 can also act as costimulatory receptors potentiating CD3/CD28-medited T-cell activation [65], a function that could be potentiated by sEVs.

TH17 cell-derived exosomes did not differ much from TH1 or TH2 cell-derived sEVs, suggesting an intermediate status between those TH-cell subsets. We could only find one protein upregulated in TH17-derived sEVs, named NAMPT. This enzyme is key in the conversion of NAM to NMN in the salvage pathway of NAD synthesis and can be released (eNAMPT or visfatin) to act as a cytokine that induces the production of pro-inflammatory (e.g., IL-6, TNF-α, CXCL8, CCL-20) and angiogenic factors (e.g., VEGF, MMP-2). NAMPT can be released by keratinocytes and dermal fibroblasts from psoriatic skin lesions [66], resulting in an augmented angiogenesis, as well as higher recruitment and activation of inflammatory cells. We have found that sEVs from TH17 cells, major players in psoriasis, are enriched in NAMPT and could potentially contribute to this pro-inflammatory microenvironment. Indeed, NAMPT gene expression was previously found upregulated in PBMCs from patients with severe psoriasis [67]. In agreement, eNAMPT was also found elevated in patients with TH17-driven pathologies, including RA [68] or IBD [69], where levels correlate with worse prognosis.

We can therefore conclude that our panel of biomarkers of sEVs produced by eosinophils, neutrophils, and different TH subpopulations may be of great clinical utility. As a proof-of-concept, sEVs from serum samples of T2^high^ asthma patients tend to present higher levels of TH2-related proteins (ITGA4 and ITGB2) than the T2^low^ counterparts, as expected.

However, like any other study, this one has some limitations. First, sEV isolation by ultracentrifugation usually results in co-purification of highly abundant serum proteins and lipoproteins [70]. Despite a thorough washing process of the sEVs during ultracentrifugation, our proteomic data still reveal contamination by serum proteins (e.g., ALB, A2M, HP, or APOA1), specially on granulocyte-delivered sEVs. To avoid bias in differential abundance studies, we have removed the highly abundant serum proteins from the analyses prior to normalising all proteins by the total sum of remaining sEV proteins. In addition, it was reported that frequently used sEV markers (such as CD9, CD81, or CD63) as well as typically used house-keeping proteins (e.g., GAPDH or vinculin) vary among sEVs delivered from different cell types [71,72]. This fact and the low sEV protein recovery made it impossible to find any loading control for western blot analysis to compare granulocyte- vs. TH-derived sEVs.

## 4. Materials and Methods

### 4.1. Bioethics and Funding

The present work was supported by the Carlos III Health Research Fund under Grant PI17/01655; miRNAs and exosome proteome in patients with asthma (microProtExAs). The research was approved by the Santiago-Lugo Research Ethics Committee on 21/06/18 (registration code 2018/125) and all study participants signed an informed consent form prior to sample collection, abiding by the Declaration of Helsinki principles. Peripheral blood samples were collected from 149 donors (18–83 years old), including healthy controls (HC) (N = 30) and asthma (N = 119) patients. All samples were collected between October-2018 and October-2020 at the Pneumology Unit of the University Hospital Complex of Santiago de Compostela (Galicia, Spain). Patients were classified according to their T2^high^/T2^low^ phenotype (T2^high^, total IgE levels ≥ 100 IU/mL, blood eosinophil count ≥ 300/mL, FeNO ≥ 30) [73]. Demographic, clinical, biochemical, and haematological variables were described in our previous publications [5,20] and in Supplementary Table S10, splitting patients into T2^low^ and T2^high^ molecular phenotype.

### 4.2. Isolation of Human Peripheral Blood Eosinophils

Eosinophils from healthy donors were isolated from 30 mL of human venous peripheral blood samples collected in K3E tubes. First, blood was incubated for 30 min with dextran (Sigma-Aldrich, St. Louis, MO, USA) (62.5 µL per mL of blood) to remove erythrocytes. The leukocyte-rich upper fraction was collected and layered on top of Histopaque^®^ 1077 (Sigma-Aldrich, St. Louis, MO, USA). The gradient was centrifuged at 400× *g* (30 min, RT, brake off) and the white band containing peripheral blood mononuclear cells (PBMCs) was discarded. Cells were washed twice with 10 mL EasySep Buffer (StemCell^TM^ Technologies, Vancouver, BC, Canada) and centrifuged at 300× *g* (10 min, 4 °C). Cell density and viability (propidium iodide; EMD Millipore, Danvers, MA, USA) were estimated using a Beckman Coulter Cytoflex flow cytometer. Cell density was adjusted at 5 × 10^7^ cells/mL in EasySep Buffer, and eosinophils were isolated with the EasySep^TM^ Human Eosinophil Isolation Kit (StemCell^TM^ Technologies, Vancouver, BC, Canada) following manufacturer instructions. Finally, eosinophil density was adjusted to 5 × 10^6^ cells/mL in a serum-free RPMI-1640 medium (Sigma-Aldrich, St. Louis, MO, USA).

### 4.3. Isolation of Human Peripheral Blood Neutrophils

Neutrophils were isolated from 12 mL of human venous peripheral blood samples collected in K3E tubes from healthy donors. After PBMC removal using Histopaque^®^ 1077 as described above, a granulocyte pellet (mostly neutrophils) was collected from the bottom of the tube and washed with 10 mL of RPMI-1640 medium (300× *g*, 10 min, 4 °C). Erythrocytes were then removed and incubated with Ammonium–Chloride–Potassium (ACK) lysis buffer (NH_4_Cl 150 mM, KHCO_3_ 10 mM, Na_2_EDTA 0.1 mM, pH 7.2–7.4) on ice for 15 min. The remaining cells were washed twice with RPMI-1460 medium and cell viability/density calculated as stated before.

### 4.4. Isolation and Differentiation of CD4^+^ T Cells

PBMCs were isolated from leukocyte concentrates (buffy coats; BCs) donated by the Organ and Blood Donation Agency (ADOS; Santiago de Compostela, Spain) and purified by a Histopaque^®^ 1077 gradient as described [74]. In brief, BCs were 1:4 diluted in RPMI-1460 and layered on top of Histopaque^®^ 1077; after centrifugation (400× *g*, 30 min, RT, brake off), the white band with the cells of interest was collected and washed twice with RPMI-1460 (350× *g*, 7 min, RT). Cell density and viability were assessed using propidium iodide (50 μg/mL) in a Cytoflex Flow Cytometer (Beckman Coulter, Brea, CA, USA). Cell density was adjusted to 5 × 10^7^ cells/mL in Isolation Buffer (Ca^2+^ and Mg^2+^ free PBS, 2% FBS, 0.09% sodium azide). Total CD4^+^ T cells were isolated using an EasySep^TM^ Human CD4^+^ T Cell Isolation Kit (StemCell^TM^ Technologies, Vancouver, BC, Canada) following manufacturer instructions.

Total CD4^+^ T cells were subsequently differentiated in vitro into TH1, TH2, or TH17 subsets. T75 flasks were pre-coated with anti-CD3 (Biolegend, San Diego, CA, USA; 37 °C, 2 h) and diluted in PBS pH 7.4 at a final concentration of 1 μg/mL (for TH1 e TH2) or 3 μg/mL (for TH17). After removing the unbound antibody by washing twice with sterile PBS at pH 7.4, a total of 25 × 10^6^ CD4^+^ T cells were seeded at 5 × 10^5^ cells/mL in complete RPMI-1640 medium (supplemented with 10% FBS, 100 IU/mL penicillin, 100 μg/mL streptomycin) and treated with 1 µg/mL (for TH1) or 3 µg/mL (for TH2 and TH17) of soluble anti-CD28 (Biolegend, San Diego, CA, USA). After activation, cells were differentiated with specific cytokines (Peprotech, Cranbury, NJ, USA) and neutralising antibodies (Biolegend, San Diego, CA, USA) for 7 days at 37 °C: For TH1 cells: IL-2 (200 IU/mL), IL-12 (10 ng/mL), IL-27 (50 ng/mL), IFNγ (100 IU/mL), and anti-IL-4 (1 µg/mL); For TH2 cells: IL-2 (200 IU/mL), IL-4 (10 ng/mL), and anti-IFNγ (1 µg/mL); For TH17 cells: IL-6 (30 ng/mL), IL-1β (50 ng/mL), IL-23 (50 ng/mL), anti-IFNγ (1 µg/mL), and anti-IL-4 (1 µg/mL). The culture medium was changed, when necessary, by replacing the repolarisation stimulus. For TH2 differentiation conditions, cell density was readjusted at day 5 and plate-bound anti-CD3 was removed. At day 7, cells were washed twice with RPMI-1460 medium to remove serum proteins and cell density was finally adjusted to 5 × 10^6^ cells/mL.

### 4.5. Characterisation of the Isolated Leukocyte Subpopulations

First, isolated cells were characterised using cytospin and eosin-methylene staining. Thirty thousand cells were adhered to slides using CytoSpin^TM^ (Thermo Shandon Cytospin3 Centrifuge, Marshall Scientific, Hampton, NH, USA; 72× *g*, 5 min). Then, slides were air dried for 5 min and cells were fixed, permeabilised, and stained (eosin/methylene-blue) by using a Kwik-Diff Stain Kit (Shandon; 30 s with each reactive). Slides were washed with distilled water and visualised using an optic microscope (CHK2-F-GS, Olympus, Tokyo, Japan).

Cell purity was also assessed using flow cytometry (FC). A total of 1 × 10^4^ cells per tube were washed twice (300× *g*, 5 min, 4 °C) with cold stain buffer (PBS pH 7.4, 1% BSA, 0.05% sodium azide, 1 mM EDTA). The supernatant was discarded and cells were incubated for 20 min at 4 °C in the dark with the following antibodies: isotypes FITC Mouse IgG1 κ (20 µL, BD BioScience, Franklin Lakes, NJ, USA), PE Mouse IgG1 κ (20 µL, BD BioScience, Franklin Lakes, NJ, USA), and PerCP Cy 5.5 Mouse IgG1 κ (20 µL, BD BioScience, Franklin Lakes, NJ, USA), and specific anti-CCR3 PerCP Cy5.5 (5 µL, BD Pharmingen^TM^, Franklin Lakes, NJ, USA), anti-CD16 PE (20 µL, BD Pharmingen^TM^, Franklin Lakes, NJ, USA), anti-CD3 FITC (20 µL, BD Pharmingen^TM^, Franklin Lakes, NJ, USA), and anti-CD4 FITC (20 µL, BD Pharmingen^TM^, Franklin Lakes, NJ, USA) antibodies. Cells were then washed twice, resuspended in 300 µL of stain buffer, and acquired (5000 events/tube) on a CytoFlex flow cytometer (Beckman Coulter, Brea, CA, USA).

CD4^+^ T-cell differentiation was also evaluated using a Luminex multiplex immunoassay (R&D Systems, Abingdon, UK). Briefly, the levels of TH1 (IFN-γ), TH2 (IL-4, IL-5, IL-10, IL-13), and TH17 (IL-1 β, IL-17) cytokines were analysed in cell-free supernatants from CD4^+^ T lymphocytes activated in vitro for 3 h with 50 ng/mL of phorbol 12-miristato 13-acetato (PMA; Sigma-Aldrich, St. Louis, MO, USA) and 1 µM of ionomycin (Sigma-Aldrich, St. Louis, MO, USA). We used a Luminex immunoassay according to the manufacturer’s instructions, and data were acquired on a Luminex MAGPIX instrument (R&D Systems, Abingdon, UK).

### 4.6. Stimulation of Small Extracellular Vesicle (sEV) Production In Vitro

sEV production from the different leukocyte subsets (5 × 10^6^ cells/mL) was performed in vitro using 50 ng/mL of PMA and 1 µM of ionomycin in a serum-free medium at different time points: 30 min for eosinophils, 1 h for neutrophils, and 3 h for the different TH-cell subsets.

### 4.7. Isolation and Characterisation of Small Extracellular Vesicles (sEVs)

sEVs were purified from pre-conditioned media by ultracentrifugation (UC) as previously described [75]. In brief, pre-conditioned media were centrifuged (10 min, 300× *g*, 4 °C) and the supernatant transferred into clean microfuge tubes. Then, tubes were sequentially centrifuged for 30 min at 2000× *g* and 10,000× *g* (4 °C). Precleared supernatant was transferred into Ultra-Clear^TM^ tubes (13 × 51 mm; Beckman Coulter) and ultracentrifuged (100,000× *g*, 4 °C, 120 min). Finally, sEVs were washed with 5 mL of 0.2 μm-filtered PBS (100,000× *g*, 60 min, 4 °C). The sEV fraction was finally resuspended in 50–200 μL PBS and stored at −80 °C.

We used transmission electron microscopy (TEM) for characterising the size and shape of sEVs, as previously described [5]. In brief, fractions of sEVs were fixed with glutaraldehyde solution, stained with phosphotungstic acid on carbon-coated copper grids, and subsequently analysed in a JEOL JEM-1011 microscope (JEOL, Inc., Tokyo, Japan) operating at an accelerating voltage of 40–100 kV. Photographs were taken with a MEGA VIEW III digital camera (SIS). All analyses were performed at the Research and Technological Development Support Infrastructure Network (RIAIDT) of the University of Santiago de Compostela, Electron and Confocal Microscopy Service, Lugo, Galicia, Spain. The size distribution of sEVs was also confirmed by dynamic light scattering (DLS) using a Zetasizer Nano ZS laser light scattering instrument (Malvern Panalytical, Malvern, UK), as previously described [5].

sEVs were lysed with radioimmunoprecipitation assay (RIPA) buffer: 25 mM Tris HCl, pH 8.0, 150 mM NaCl, 1% NP-40, 1% sodium deoxycholate, 0.1% SDS. After a vigorous vortex, lysis was allowed for 30 min at 4 °C, followed by sonication in an ice bath for 30 s and further incubation on ice and occasional shaking for 15 min. Protein concentration was determined using Pierce™ BCA Protein Assay Kit (ThermoFisher Scientific^®^, Bedford, MA, USA) following manufacturer instructions. Absorbance was recorded at 550 nm in a Multiskan^TM^ Microplate Reader (ThermoFisher Scientific^®^, Bedford, MA, USA). sEV proteins were finally stored at −80 °C before further analyses.

### 4.8. Qualitative and Quantitative Proteomics Analysis

The analysis was performed as previously described [76,77]. In brief, 15 μg of sEV proteins were concentrated (60 V) in a resolving 10% SDS-PAGE gel and the protein band was stained (Sypro Ruby fluorescent staining; Lonza, Basel, Switzerland) and excised. Gel pieces were reduced (10 mM DTT; Sigma-Aldrich, St. Louis, MO, USA) and alkylated (55 mM iodoacetamide; Sigma-Aldrich, St. Louis, MO, USA). Then, we performed in-gel tryptic digestion, as described elsewhere [78]. Peptides were extracted [50% ACN/0.1% TFA (×3) and ACN (×1)], pooled, concentrated in a SpeedVac, and stored at −20 °C.

For qualitative analysis, triplicates from each sample were pooled, and 4 µg of the 5 resulting samples (neutrophils, eosinophils, TH1, TH2, TH17) were separated by reverse-phase liquid column chromatography (LC) (Nano-LC ultra-System Eksigent) and analysed on an Eksigent TripleTOF^®^ 6600 system (Sciex, Redwood City, CA, USA) using a data-dependent acquisition (DDA) method, as previously described by our group [76]. Peptides and proteins were identified with the Protein Pilot^TM^ software (version 5.0.1; Sciex, Redwood City, CA, USA) and the human UNIPROT database, with methionine oxidation as the variable modification and cysteine carbamidomethylation as the fixed modification. A false discovery rate (FDR) of 1% was used. Proteins exclusively detected in certain sEV samples and those shared by different sEV types were identified with the Scaffold software (version 5.1.2, Proteome Software Inc., Portland, OR, USA; accessed on 3 September 2022).

For label-free quantitative proteomics, we performed an MS analysis by sequential window acquisition of all theoretical mass spectra (SWATH-MS), as previously described [76]. First, a unique peptide pool was created by mixing equal amounts of peptides from each sample (neutrophils, eosinophils, TH1, TH2, TH17). This pool was analysed by triplicate by LC-MS/MS on a TripleTOF^®^ 6600 LC-MS/MS system via a data-dependent acquisition (DDA) method to create a SWATH-MS spectral library [76,77]. This spectral library contained the retention time and the spectral data of both parental peptides (MS1) and fragmentation (MS2) spectra. Then, 4 µg of peptides derived from sEVs produced by neutrophils, eosinophils, or lymphocytes (TH1, TH2, TH17) were individually analysed by SWATH-MS. SWATH-MS acquisition was performed on a TripleTOF^®^ 6600 LC-MS/MS system via a data-independent acquisition (DIA) method. The whole 400 to 1250 m/z range was covered in 100 steps with spectral windows of variable width (1 m/z overlap). Peaks extraction was carried out with PeakView software (version 2.2; Sciex, Redwood City, CA, USA) and scored using the PeakView SWATH Acquistion MicroApp (version 2.0; Sciex, Redwood City, CA, USA). The integrated peak areas were exported to the MarkerView software (version 1.3, Sciex, Redwood City, CA, USA; accessed on 3 September 2022). To ensure a more accurate comparison between samples, well-known endogenous peptides were used during data alignment to compensate for small variations in both mass and retention times. The amount of each protein in every sEV sample was calculated as the averaged area sums of 10 peptides per protein and 7 transitions per peptide. Then, an averaged MS peak area of each protein was calculated. Data normalisation was carried out with the most likely ratio normalisation (MLR) method. As part of the initial analysis, the MarkerView software (version 1.3, Sciex, Redwood City, CA, USA) also allowed a principal component analysis (PCA) to see how well each protein distinguishes between groups. Proteomic data are available at ProteomeXchange under accession number PXD046108.

### 4.9. Western Blot Assays

Two to ten μg (indicated at each figure) of sEV protein were resuspended in 1X SDS-PAGE loading buffer (62.5 mM Tris-Cl pH 6.8, 2.5% SDS, 12.5% glycerol, 0.1% bromophenol blue, 5% β-mercaptoethanol). Samples were boiled at 95 °C for 5 min and electrophoresed on precast 4–20% SDS-PAGE gels (Bio-Rad, Hercules, CA, USA) at 150 V in a Mini-PROTEAN^®^ 3 Cell (Bio-Rad, Hercules, CA, USA) with a 1X Tris/Glycine/SDS buffer (Bio-Rad, Hercules, CA, USA) and 5 μL of pre-stained protein ladder Precision Plus Protein^TM^ WesternC^TM^ (Bio-Rad, Hercules, CA, USA). Proteins were transferred to 0.2 μm mini-size nitrocellulose membranes (Bio-Rad, Hercules, CA, USA) using a Trans-Blot^®^ Turbo system (Bio-Rad, Hercules, CA, USA) and 1X Trans-Blot Turbo transfer buffer (Bio-Rad, Hercules, CA, USA). The membrane was blocked for 2 h at RT with 5% bovine serum albumin (BSA) in TBS-T (20 mM Tris HCl pH 7.6, 150 mM NaCl, 0.1% Tween-20). Then, the membrane was washed twice with TBS-T and incubated overnight (4 °C) with the following primary antibodies diluted in the blocking solution: anti-GAPDH (1:2000; ab37168; abcam, Cambridge, UK), anti-ISG15 (1:5000; 15981-1-AP; proteintech, Rosemont, IL, USA), anti-ITGA4 (1:2000; 19676-1-AP; proteintech, Rosemont, IL, USA), anti-ITGB1 (1:1000; sc-18887; Santa Cruz, Dallas, TX, USA), anti-PBEF/NAMPT (1:1000; 11776-1-AP; proteintech, Rosemont, IL, USA), anti-LTF (1:500; sc-52048; Santa Cruz, Dallas, TX, USA), anti-NF45/ILF2 (1:500; sc-365283; Santa Cruz, Dallas, TX, USA), anti-NGAL/LCN2 (1:500; sc-518095; Santa Cruz, Dallas, TX, USA), and anti-RPL13 (1:500; sc-100829; Santa Cruz, Dallas, TX, USA). The membrane was washed five times with TBS-T (5 min/each) and incubated for 2 h at RT with secondary antibodies: anti-rabbit IgG HRP-linked (1:20,000; 4010-05; Southerbiotech, Homewood, AL, USA) or anti-rabbit IgG HRP-linked (1:3000; 7074S; Cell Signaling Tecnhologies, Danvers, MA, USA); and anti-mouse IgG HRP-linked (1:20,000; 1031-05; Southerbiotech, Homewood, AL, USA) or anti-mouse IgG HRP-linked (1:3000; 7076S; Cell Signaling Technology, Danvers, MA, USA). Detection was performed by chemiluminescence (AR1170 kit, Boster Bio, Pleasanton, CA, USA) and immunoblots were visualized in a ChemiDoc Imaging System (Bio-Rad, Hercules, CA, USA).

### 4.10. Bioinformatic Analysis and Statistics

For qualitative proteomic data and the enrichment analyses of Gene Ontology (GO) terms, we have used the Functional Enrichment analysis tool (FunRich_3.1.3 software), selecting the top 10 or top 6 enriched biological processes (BP).

For quantitative proteomic analyses, multiple *t*-test analyses were performed and proteins with *p* < 0.05 and a log2 fold-change (FC) > 1 were considered differentially abundant. Volcano plots were represented using the R package EnhancedVolcano. Gene set enrichment analyses (GSEA) of Gene Ontology (GO) of identified proteins were performed using the gseGO function of the ClusterProfiler package in R studio. Biological process (BP) categories were analysed. Representations of enriched terms were performed using the dotplot and cnetplot (gene–concept network) functions from the enrichplot package in R studio. Enrichment analyses for KEGG and Reactome pathways, and protein–protein interaction networks were performed using the STRING database (v.12.0 database, free access at http://string-db.org (accessed on 7 September 2023)). Statistical analyses of comparisons between more than one group were performed using one-way analysis of variance (ANOVA) followed by the Holm–Šídák multiple comparisons test.

## 5. Conclusions

Our results demonstrate that the proteome of sEVs, delivered by different immune cell populations involved in chronic inflammatory diseases, is variable and highly related to the cellular origin. These signature sEV proteins are found in serum and can potentially be used to define asthma phenotypes. Therefore, we have described a set of immune-related sEV proteins that can serve as non-invasive and clinically accessible biomarkers of disease. This is particularly important in chronic inflammatory disorders, such as asthma and COPD, where these specific inflammatory pathways are predominant.

## Data Availability

Proteomic data are available at ProteomeXchange under accession number PXD046108.

## Supplementary Materials

The supporting information can be downloaded at https://www.mdpi.com/article/10.3390/ijms251910848/s1.
