# Supplementary material for: Signature Proteins in Small Extracellular Vesicles of Granulocytes and CD4+ T-Cell Subpopulations Identified by Comparative Proteomic Analysis
Source: Int J Mol Sci. 2024 Oct 9;25(19):10848. doi: 10.3390/ijms251910848 (PMC11476868; doi:10.3390/ijms251910848)
Supplement: Supplementary file 1 [file ijms-25-10848-s001.zip › Supplementary Figures.pdf]

Title:

Signature Proteins in Small Extracellular Vesicles of Granulocytes and $CD4^+$ T Cell Subpopulations Identified by Comparative Proteomic Analysis

Authors:

Sara Vázquez-Mera, Pablo Miguéns-Suárez, Laura Martelo-Vidal, Sara Rivas-López, Lena Uller, Susana Belén Bravo, Vicente Domínguez Arca, Xavier Muñoz, Francisco Javier González-Barcala*, Juan José Nieto Fontarigo, Francisco Javier Salgado.

González-Barcala FJ and Nieto-Fontarigo JJ have co-directed the work.

Supplementary Figures

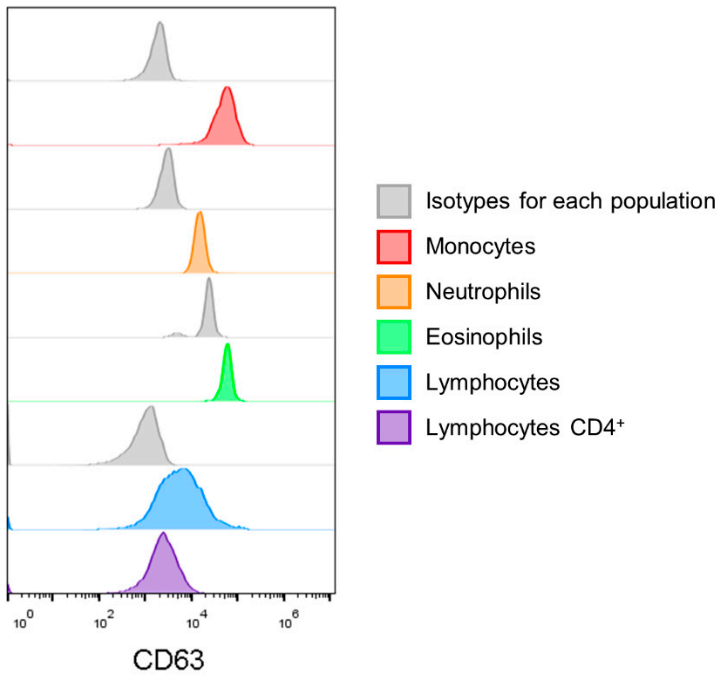

Supplementary Figure S1. Surface levels of CD63 in different leukocyte populations measured by flow cytometry.

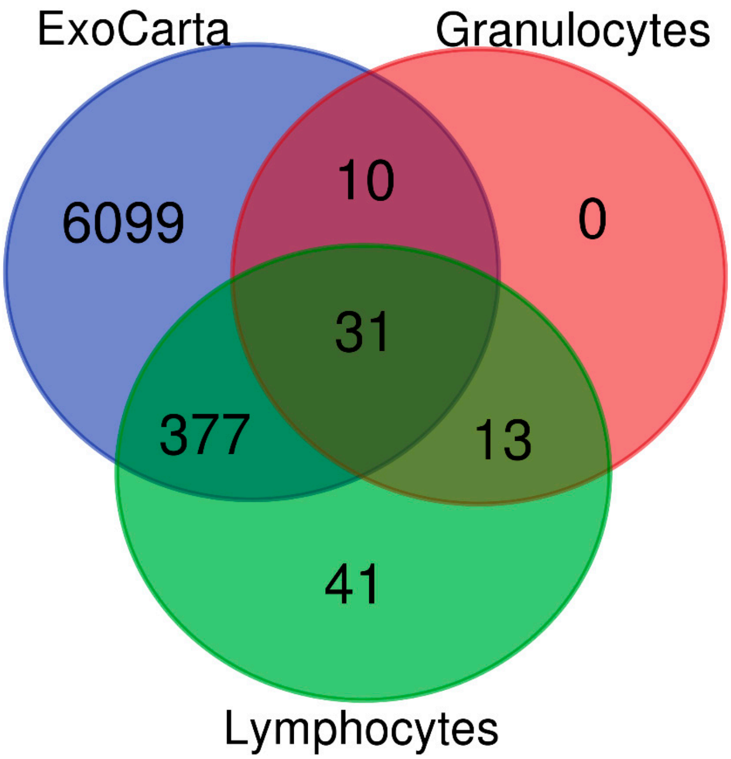

Supplementary Figure S2. Venn diagrams of identified proteins in lymphocyte- and granulocyte-derived exosomes that are present in Exocarta database.

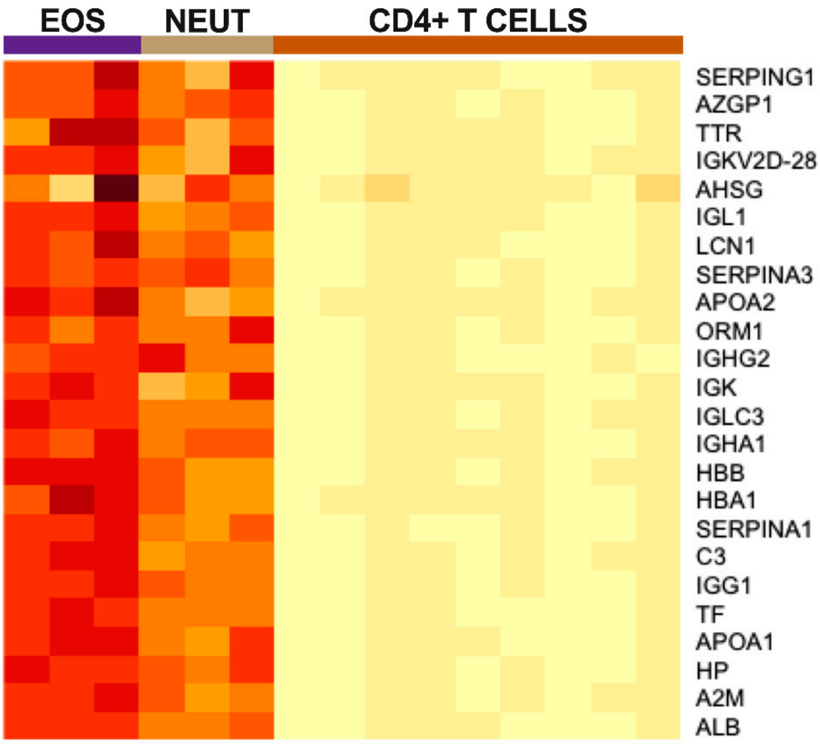

Supplementary Figure S3. Changes in high abundant serum proteins between sample groups. A) Heatmap plot with levels of abundant serum proteins (N=24) found in the different sample groups.

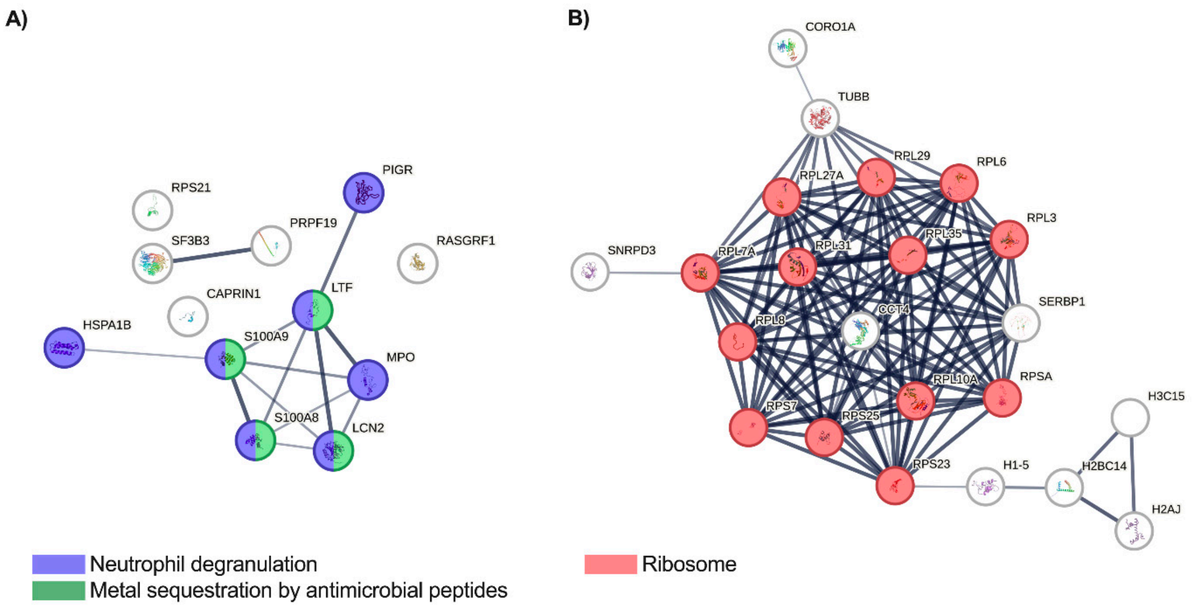

Supplementary Figure S4. Pathways enriched in granulocyte and TH cell derived exosomes. A) Network analysis of proteins highly upregulated in granulocytes (fold-change>3) using String database. **B)** Network enrichment analysis of proteins highly upregulated in TH cells (fold-change> 2) using String database. Relevant enriched pathways are highlighted.

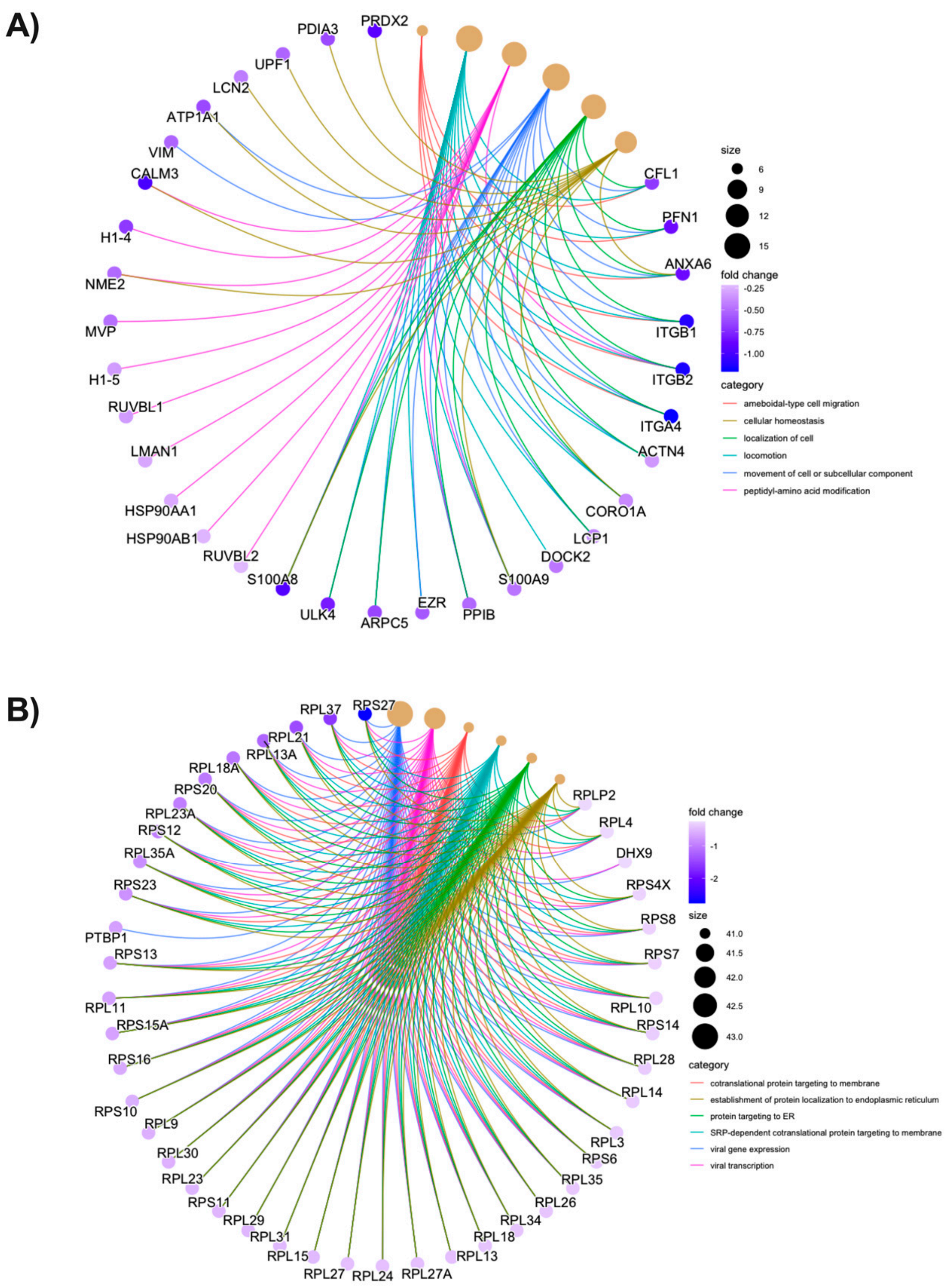

Supplementary Figure S5. Gene Set Enrichment Analyses (GSEA) of sEV proteins from TH1 and TH2 cells. Category netplot of the 6th most down-regulated pathways in TH1 **(A)** and TH2 **(B)** cell-derived sEV.
